# Low glycemic index diet restrains epileptogenesis in a gender-specific fashion

**DOI:** 10.1007/s00018-023-04988-1

**Published:** 2023-11-10

**Authors:** Caterina Michetti, Daniele Ferrante, Barbara Parisi, Lorenzo Ciano, Cosimo Prestigio, Silvia Casagrande, Sergio Martinoia, Fabio Terranova, Enrico Millo, Pierluigi Valente, Silvia Giovedi’, Fabio Benfenati, Pietro Baldelli

**Affiliations:** 1 Department of Experimental Medicine, University of Genova, Genoa, Italy; 2 Department of Informatics, Bioengineering, Robotics and System Engineering, University of Genova, Genoa, Italy; 3 Center for Synaptic Neuroscience and Technology, Italian Institute of Technology, Genoa, Italy; 4 IRCCS, Ospedale Policlinico San Martino, Genoa, Italy

**Keywords:** Epileptiform activity, Glycemia, Ketogenic diet, Temporal lobe epilepsy, Tonic GABAergic inhibition, Sex effect, 3α,5α-tetraidroprogesterone, Syn2, Tonic–clonic

## Abstract

Dietary restriction, such as low glycemic index diet (LGID), have been successfully used to treat drug-resistant epilepsy. However, if such diet could also counteract antiepileptogenesis is still unclear. Here, we investigated whether the administration of LGID during the latent pre-epileptic period, prevents or delays the appearance of the overt epileptic phenotype. To this aim, we used the Synapsin II knockout (SynIIKO) mouse, a model of temporal lobe epilepsy in which seizures manifest 2–3 months after birth, offering a temporal window in which LGID may affect epileptogenesis. Pregnant SynIIKO mice were fed with either LGID or standard diet during gestation and lactation. Both diets were maintained in weaned mice up to 5 months of age. LGID delayed the seizure onset and induced a reduction of seizures severity only in female SynIIKO mice. In parallel with the epileptic phenotype, high-density multielectrode array recordings revealed a reduction of frequency, amplitude, duration, velocity of propagation and spread of interictal events by LGID in the hippocampus of SynIIKO females, but not mutant males, confirming the gender-specific effect. ELISA-based analysis revealed that LGID increased cortico-hippocampal allopregnanolone (ALLO) levels only in females, while it was unable to affect ALLO plasma concentrations in either sex. The results indicate that the gender-specific interference of LGID with the epileptogenic process can be ascribed to a gender-specific increase in cortical ALLO, a neurosteroid known to strengthen GABAergic transmission. The study highlights the possibility of developing a personalized gender-based therapy for temporal lobe epilepsy.

**Supplementary Information:**

The online version contains supplementary material available at 10.1007/s00018-023-04988-1.

## Introduction

Epilepsy is one of the most common and serious chronic neurological diseases, affecting 1% of the world population and characterized by spontaneous recurrent seizures. Seizures are the epiphenomenon of modifications of neural circuits that, starting from an initial insult, become hyperexcitable during a latent process, named epileptogenesis. The initial insult activates multiple and complex cascades of events, lasting from hours to months, encompassing neurodegeneration, inflammatory activity, transcriptional events, neurogenesis, sprouting, reorganization of neuronal circuits and gliosis [1, 2]. Using maladaptive mechanisms proper of neural plasticity, the epileptogenic process progressively alters neuronal excitability and modifies circuit connectivity before the first seizure occurs [3]. In spite of the availability of a large toolbox of antiepileptic drugs (AEDs) capable of suppressing seizures, an anti-epileptogenic (AEG) strategy able to efficiently contrast the epileptogenic process and prevent epilepsy is still on demand [4].

In the last 2 decades, several clinical trials based on the use of conventional AEDs for preventing epilepsy have been carried out, but the results have been unsuccessful or controversial, so that effective AEG drugs for treating people at risk are still missing [4, 5]. A possible explanation for this failure is that the molecular mechanisms of epileptogenesis differ from those of seizure manifestation. Seizures are a product of an overt excitatory/inhibitory imbalance, while epileptogenesis represents a complex cascade of events leading to the imbalance condition. AEDs act attempting to recover the balance producing an opposite imbalance elsewhere in the brain. On the contrary, to contrast epileptogenesis, it is probably more efficient a physiological strengthening of intrinsic homeostatic processes that normally defend neuronal networks from brains insults [6, 7]. Moreover, a preventive treatment for epilepsy with AEDs must be considered with caution in vulnerable patients showing markers of epileptogenesis associated with moderate probability of developing seizures, as in the case of cerebral malaria in which only 10% of children presenting clear electrographic signs will develop epilepsy [8]. Thus, in people with uncertain probability of developing epilepsy, the risk of side effects due to treatment with conventional AEDs has to be considered, raising serious doubts on the opportunity of a treatment for preventive/protective purposes [4].

Metabolism-based therapies such as the traditional high-fat, low-carbohydrate ketogenic diet (KD) and the much better tolerated, low-glycemic index diet (LGID) [9–11] may represent an alternative to prevent the development of epilepsy, because of their proven success in arresting drug-resistant seizures [12, 13] coupled with the relative absence of side effects that allows chronic treatments in patients with uncertain risk of epilepsy, even in the pediatric age. More importantly, a large body of experimental evidence demonstrates that diet-based treatments activate multiple homeostatic mechanisms in the brain to increase seizure threshold [14–16] and make neuronal networks more resilient to stressors [17], thus preventing or reversing seizure progression [18–20]. Here, we studied the efficacy of LGDI in reverting the epileptogenic process in the Synapsin II knockout (SynIIKO) mice, an experimental model of monogenic reflex temporal lobe epilepsy caused by dysfunctions of synaptic transmission.

The synapsins are a family of neuron-specific phosphoproteins encoded by three distinct genes (*Syn1, Syn2 and Syn3*) that are abundantly expressed in the brain and concentrated in synaptic terminals where they act by regulating neurotransmitter release [21–25]. Large-scale search for genetic susceptibility loci in epilepsy identified *SYN2* as one of the five major genes that contribute to epilepsy predisposition in humans [26]. These findings were supported by subsequent studies, revealing that polymorphisms in *SYN2* are associated with idiopathic generalized epilepsy [27–29]. Both SynIKO and SynIIKO mice show an epileptic phenotype, consisting of partial secondarily generalized tonic–clonic seizures, that is more severe in SynIIKO mice or in double SynI/SynIIKO mice [30–33]. Importantly, the behavioral seizures in SynIIKO mice are characterized by a late onset, thus offering an operational window to test whether LGID modifies the epileptogenic process.

In the present work, SynIIKO mice fed with either LGID or standard diet (StD) during gestation and the postnatal life up to 5 months of age were investigated behaviorally for the appearance of epilepsy and electrophysiologically for the presence of interictal events in acute cortico-hippocampal slices. Behavioral and electrophysiological results revealed protective effects of LGID only in SynIIKO females. ELISA-based analysis revealed that LGID-fed female mice had higher cortical level of allopregnanolone (ALLO), a neurosteroid known as an agonist on GABAergic transmission, providing a mechanistic basis for the peculiar gender-specific effect of LGID in this mouse model of temporal lobe epilepsy.

## Materials and methods

### Animals and diets

SynIIKO mice were generated by homologous recombination and extensively backcrossed on a C57BL/6 J background (Charles River, Calco, Italy) for over 10 generations [30, 34, 35]. Each homozygous SynIIKO female mouse was housed with one homozygous SynIIKO male in standard Plexiglas cages (33 × 13 cm), with sawdust bedding and a metal top. Female SynIIKO mice were split into two groups and fed ad libitum either StD or LGID starting from mating [36]). The two diets (Bio Serv, Flemington, NJ) were isocaloric (3.7 kcal/g), with the same nutritional profile (carbohydrate 65%, protein 20%, fat 5%, fiber 5%, moisture 5%) and identical in micro- and macro-nutrients except for the type of starch, representing the main source of carbohydrate (Supplementary Table 1). The starch in the LGID was a combination of 70% amylose and 30% amylopectin (Hylon VII starch; Ingredion, Westchester, IL), whereas the starch in the StD was 100% amylopectin (Amioca starch; Ingredion, Westchester, IL).

**Fig. 1:**
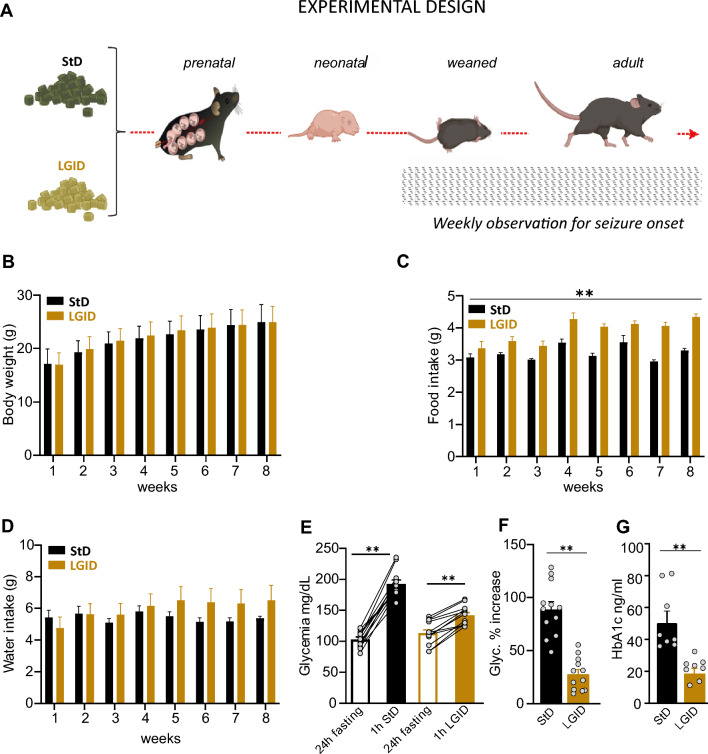
Experimental plan and parameters analyzed during the administration of the two diets. **A** StD and LGID were delivered ad libitum to SynIIKO pregnant females starting from mating and continuing during pregnancy and lactation. Mice were maintained with LGID also after weaning and for the entire lifespan. Starting from the day of weaning, all mice were weekly checked for two months for body weight (**B**), food intake (**C**) and water consumption (**D**). Parameters analyzed in the two experimental groups revealed that LGID mice increased food consumption, although no differences appeared in body weight and water consumption (*n* = 30 KO StD; *n* = 32 KO LGID). Five months after birth all mice were subjected to blood analysis for the evaluation of glycemia (**E**, **F**) and glycated hemoglobin (**G**) levels. **E** For the analyses of blood glucose, mice were fasted for 24 h. Glycemia was measured immediately after the fasting period and 1 h after food administration. Glycemia levels showed a increase after food consumption in StD and LGID-fed mice (*n* = 12 for both StD and LGID). **F** Percentage increase of glycaemia observed 1 h after food administration (*n* = 12 for both StD and LGID). **G** Glycated hemoglobin levels were higher in StD-fed mice when compared with animals treated with LGID (*n* = 8 for both StD and LGID). Data are expressed as means ± sem. *p < 0.05, **p < 0.01; two-way repeated-measures ANOVA (**B**–**D**), paired Student’s *t*-test (**E**), unpaired Student's *t*-test (**F**), unpaired Mann–Whitney *U*-test (**G**)

SynIIKO pregnant and lactating mothers and pups therefrom were fed ad libitum with either diet (Fig. 1A). After weaning, male (M) and female (F) offsprings of each group of mothers were kept on the same diets as their mothers (*n* = 16 M StD, *n* = 21 M LGID, *n* = 14 F StD; *n* = 12 F LGID). Sample size was chosen based on previous experience with behavioral seizures in SynIIKO mice [31]. Starting from weaning (P25), each mouse was weekly checked for body weight, food intake, water consumption and seizure onset. Blood glucose and glycated hemoglobin were assayed at 5 months of age (Fig. 1 and Supplementary Figs. 1–3). Mice were maintained on a 12∶ 12 h light/dark cycle (lights on at 7 a.m.) at constant temperature (21 ± 1 °C) and relative humidity (60 ± 10%). All experiments were carried out in accordance with the guidelines established by the European Communities Council (Directive 2010/63/EU of March 4th, 2014) and were approved by the Italian Ministry of Health (authorization n° 600/2020-PR).

### Behavioral seizures

Seizure provocations were performed every first day of the week (between 2 and 4 p.m.), starting from the day of weaning (P25), in acoustically isolated animal housing room. The provocation consisted of moving the animal from its cage to a new cage. The procedure started with opening the lid and lifting each mouse by its tail into an adjacent cage equipped with fresh bedding. This procedure is known to elicit reflex seizures in SynIIKO mice [31–33, 35, 37]. All provocations were recorded on tape with a digital camera located in front of the new cage. Video recordings were later streamed to a PC for digital storage and detailed behavioral seizure analysis were made off-line. Due to the short time interval between seizure provocation and seizure generalization, we did not apply a severity score scale, but identified the following three main elements of the seizure based on the previously described ethological description [31, 33]: (i) head and body myoclonic jerks, including retreating orofacial/forelimb twitch; (ii) whole tonic–clonic seizure (with or without jumping), including emprostoclonus, emprostotonus, epistotonus (iii) post-ictal immobility. The running fit activity was observed only in few animals and was not included in the analysis. Latency to the first observed seizure (days from the date of birth), duration of each seizure, and percent of animals jumping were analyzed. At the end of behavioral follow-up, 5-month-old animals were sacrificed to collect blood samples and brains for biochemical and electrophysiological studies.

### Brain slice preparations

All experiments were performed on symptomatic (5 months of age) SynIIKO mice of either sex. Animals were anaesthetized with isofluoran prior to decapitation, the brain was quickly dissected out and immersed in an ice-cold oxygenated “cutting solution” composed of (mM): 125 NaCl, 25 NaHCO_3_, 25 glucose, 2.5 KCl, 1.25 NaH_2_PO_4_, 1 CaCl_2_, 2 MgCl_2_, 0.4 ascorbic acid, 2 NaPyruvate, 3 myo-inositol, and saturated with 95% O_2_/5% CO_2_. Transverse hippocampal slices (300 μm thick) were cut using a Microm HM 650 V microtome equipped with a MicromCU65 cooling unit (Thermo Fisher Scientific, Waltham, MA). Slices were cut at 2 °C in a high-sucrose protective solution containing (in mM): 87 NaCl, 25 NaHCO_3_, 2.5 KCl, 0.5 CaCl_2_, 7 MgCl_2_, 25 glucose, 75 sucrose and saturated with 95% O_2_/5% CO_2_. Slices were incubated for 30–45 min at 35 °C and for at least another hour at room temperature in recording standard solution (artificial cerebrospinal fluid, ACSF) composed of (mM): 125 NaCl, 25 NaHCO_3_, 25 glucose, 2.5 KCl, 1.25 NaH_2_PO_4_, 2 CaCl_2_, 1 MgCl_2_. Prior to being used for recordings slices were pre-incubated for 20 min in recording solution supplemented with 4-aminopyridine (4-AP; 200 μM; Sigma-Aldrich, Milan, Italy). Slices were then transferred to a “submerged” high-density multielectrode array (HD-MEA) recording chamber which was continuously superfused at a rate of 1.5 ml/min with ACSF supplemented with 4-AP 200 μM. The bath temperature was monitored and maintained at 33 °C throughout the experiments.

In accordance with the rare occurrence of spontaneous seizures in vivo, slices from Syn IIKO mice showed only sporadic spontaneous epileptiform activity under control perfusion conditions. The paucity of spontaneous paroxysms is also related to slice deafferentation and the submerged modality of MEA recordings. Thus, SynIIKO slices were perfused with the K^+^ channel blocker 4-AP, a broad inhibitor of voltage-gated K_v_1–K_v_4 potassium channel subtypes [38–40], prolonging action potentials and thereby increasing neurotransmitter release at the presynaptic terminals [41]. 4-AP is widely used to cause epileptiform-like activity in in vitro and ex vivo preparations [42–45], as previously reported for SynIIKO hippocampal slices with respect to slices obtained from wild-type animals [37].

### HD-MEA recordings of spontaneous epileptiform activity in brain slices

To record electrophysiological activity in brain slices, we used the Biocam X high-density CMOS-based multielectrode arrays (HD-MEA; 3Brain AG, Switzerland). The chip integrates amplification and analog multiplexing circuits that provide simultaneous extracellular recordings from 4096 electrodes (also called pixels) at a sampling rate of 18 kHz per channel. Each square pixel measures 21 × 21 μm, and the array is integrated with an electrode pitch (center-to-center) of 81 μm. Pixels are arranged in a 64 × 64 array configuration, yielding an active area of 5.12 × 5.12 mm with a pixel density of 156.3 pixel/mm^2^. Three on-chip amplification stages provide a global gain of 60 dB, with a 0.1- to 5-kHz band-pass filter. This bandwidth is adapted to record both slow local field potentials (LFPs) and fast action potentials (APs). Acquisition was controlled using the Brain Wave software (3Brain AG, Switzerland). Acute cortico-hippocampal slices were recorded for 10 min per session, once activity had stabilized for at least 15 min. Bath application of 4-AP (200 μM) [46] favored the induction of epileptiform activity characterized by spontaneous spike-wave interictal discharges (I-ICs) that can be visualized as real-time video images in which each pixel of the video’s frames represents a recording-electrode of the array and has a color corresponding to the voltage amplitude according to a color-code map (see Supplementary Fig. 4B). Spike-wave complexes, usually lasting between 50 and 400 ms, recorded by a single recording-pixel were named I-IC waves, while groups of I-IC waves that are temporally aggregated and recorded by an ensemble of spatially clustered pixels were named I-IC events.

I-IC waves were detected by the BrainWave software (3Brain AG) that adopts a previously described Precision Timing Spike Detection (PTSD) algorithm [47, 48] originally tailored to detect fast-spiking activity generated by cultured neurons and adapted to detect slower local field potential events. To this purpose, the threshold was set to fivefold the standard deviation of the noise, whereas the refractory period and the peak lifetime period were set to 40 and 50 ms, respectively. To evaluate the effects of the LGID on I-IC waves, amplitude (maximum value of the waveform modulus), duration (time an I-IC wave takes to extinguish) and energy (time integral of the I-IC wave modulus) were computed.

I-IC events were detected with a custom program written in Pyton (v3.7.1) that analyzes I-IC waves identified by the BrainWave software. For the detection of I-IC events, we preliminarily evaluated the function H(t) representing the temporal aggregation of I-IC waves detected in each pixel-channel of a selected group (G):

$$\xi (t)\, = \,\sum\limits_{i \in G} {\xi_{i} (t)} ,$$

as follows:

$${\text{H}}(t) = \,N(t - T/2,\,t + T/2) = \,\int_{t - T/2}^{t + T/2} {\xi (\tau )} d\tau$$

where N(t_a_,t_b_) is the number of I-IC waves in the (t_a_,t_b_) interval and T the time window of integration. H(t) is computed at discrete time values t (0, ∆t, 2∆t, 3∆t…) and represents how many I-IC waves are detected within the time window centered in t. I-IC events are detected applying a threshold on H(t) values and represent ensembles of temporally and spatially related I-IC waves in a specific area of the cortico-hippocampal slice. I-IC events can be defined as an ensemble I-IC waves that are temporally and spatially related, representing the temporally synchronized activation of an aggregated of multiple neurons (i.e., recording-pixels) in a specific area of the cortico-hippocampal slice. I-IC events are tracked in time and in space, to monitor their area and rate of propagation in the slice. To evaluate the effects of the LGID on I-IC events, we extracted the following features from the recordings: (i) affected area, as the ratio between the number of activated pixels/channels recording an I-IC wave and the total number of pixels/channels covering the cortical or hippocampal area; (ii) duration, as the time difference between the last and the first I-IC wave of a detected event; frequency, as the number of I-IC events detected per min; covered distance and propagation speed, by analyzing the I-IC event propagation in space and in time. To capture the characteristics of propagation of the I-IC event occurring in the recording of the brain slice activity, a computer vision approach has been adopted. The developed algorithm is composed of two phases: video extraction and multiple object tracking (MOT). The software computes two different values: one is the average propagation speed, namely the weighted mean of the average speeds of each track, whose weights are the durations of each track trajectory; the second is the distance covered by the event, calculated as the sum of distances covered by each trajectory. All procedures used to detect and analyze I-IC events and waves were carried out blind to the experimenter thanks to algorithms based on custom programs written in Pyton (v3.7.1).

### Glycemia and glycated hemoglobin measurements

Blood glucose concentration was measured using a Mini Glucometer (Accu-Chek Aviva, Roche) by tail vein puncture of SynIIKO mice fed for 1 h with either StD or LGID after a 24 h fasting period. Glycemic index values were expressed as mg/dl of blood sample. Glycated hemoglobin (HbA1c) levels were measured in serum samples from the same animals using the HbA1c ELISA kit (mouse) (OKEH00661, Aviva System Biology, San Diego, CA) according to the manufacturer’s instructions. All standards and samples were run in duplicate. The optical density values were read at 450 nm in a multiplate reader (Tecan Infinite^®^ F500, Tecan Trading AG, Switzerland). HbA1c concentrations were expressed as ng/ml of blood sample.

### Brain allopregnanolone quantification

Cortico-hippocampal tissues from SynIIKO mice fed with either StD or LGID, were dissected after decapitation, immediately weighted and frozen in liquid nitrogen and stored at − 80 °C until analysis. All females were at their diestrus stage according to vaginal cytology. An organic phase extraction with acetonitrile to solubilize steroids and hexane to remove fat and lipids was performed. Fifty mg of frozen samples were thawed on ice and homogenized in 15 ml acetonitrile with a Teflon-glass homogenizer. After centrifugation at 10,000 × g for 10 min at 4 °C, the supernatant was carefully transferred to a clean glass tube, added with 15 ml hexane and vigorously shaken. The organic phase was collected through a separatory funnel and the fat removal step was repeated twice. Acetonitrile was evaporated to dryness under a rotary evaporator (Rotavapor^®^ R-100, BÜCHI Labortechnik AG, Switzerland) and successively in a concentrator centrifuge (VR-maxi St. a, Heto-Holten A/S, Denmark). Dried samples were frozen at − 20 °C and subsequently used for allopregnanolone (ALLO) quantification. ALLO concentrations were determined using the DetectX ALLO Immunoassay Kit (K044–H1, Arbor Assays, Ann Arbor, MI) according to the manufacturer's protocol. After solubilization of dried steroids with a volume of ethanol followed by a volume of the kit Assay Buffer in a 1:4 (v:v) ratio, samples were vigorously shaken and allowed to sit 5 min at room temperature to ensure complete steroid solubilization. Reconstituted samples were immediately immunoassayed after bringing ethanol in the sample volume below 5% with kit specific Assay Buffer. All standards and samples were run in duplicate. The optical density values were read at 450 nm in a multiplate reader (Tecan Infinite® F500). ALLO concentration values were normalized on tissue weights and reconstitution volumes and expressed as pg/mg of tissue weight.

### Statistical analysis

Data are given as means ± sem for *n* = sample size. The normal distribution of experimental data was assessed using D’Agostino-Pearson’s normality test. The *F*-test was used to compare variance between two sample groups. To compare two normally distributed sample groups, the unpaired Student’s *t*-test was used, with Welch’s correction applied in case the variance of the two groups was different. To compare two sample groups that were not normally distributed, the Mann–Whitney’s *U*-test was used. To compare more than two normally distributed sample groups, we used one-ANOVA, followed by the Tukey’s post hoc multiple comparison test. In case data were not normally distributed, one-way ANOVA was substituted with the Kruskal–Wallis’s test, followed by the Dunn’s post hoc multiple comparison test. Alpha levels for all tests were 0.5% (95% confidence intervals). Statistical analysis was carried out by using the Prism software (GraphPad Software, Inc.).

## Results

### Effects of LGID on general health and glycaemia

Breeding female SynIIKO mice were split into two groups and fed ad libitum either StD or LGID. The diet started with mating and continued during lactation and offspring were fed the same diet after weaning for their entire life span (5 months). For 8 weeks starting from the day of weaning (25d), all offspring mice (males and females) were weekly checked for body weight, food intake and water consumption (Fig. 1A–D). The measurements revealed a moderate but significant increase of food consumption in LGID-treated mice which was not accompanied by an increase in bodyweight or water consumption.

With the use of LGID, we expected to maintain low and stable levels of glycemia and to reduce glycemic peaks. For this reason, we measured glycemia and glycated hemoglobin (HbA1c) in all SynIIKO mice 5 months after birth. To investigate the effects of the two diets on the post-prandial glycemic peak, glycemia was measured after a fasting period of 24 h and 1 h after food administration that immediately followed the fasting period (Fig. 1E, F). Fasting blood glucose levels showed similar values in StD and LGID treated mice, while food intake induced a significantly higher glucose level increase (percent increase vs fasting: ~ 80%) in StD-fed mice with respect to LGID-fed mice (percent increase vs fasting: ~ 30%) (Fig. 1E, F). We also measured the levels of glycated hemoglobin A1c (HbA1c) that integrates blood glucose levels over time [49] and consistently found that LGID-treated SynIIKO mice displayed a two-fold decrease in HbAc1 levels with respect to SynIIKO mice fed with StD (Fig. 1G).

### Sex-dependent effect of LGID on behavioral seizures

**Fig. 2:**
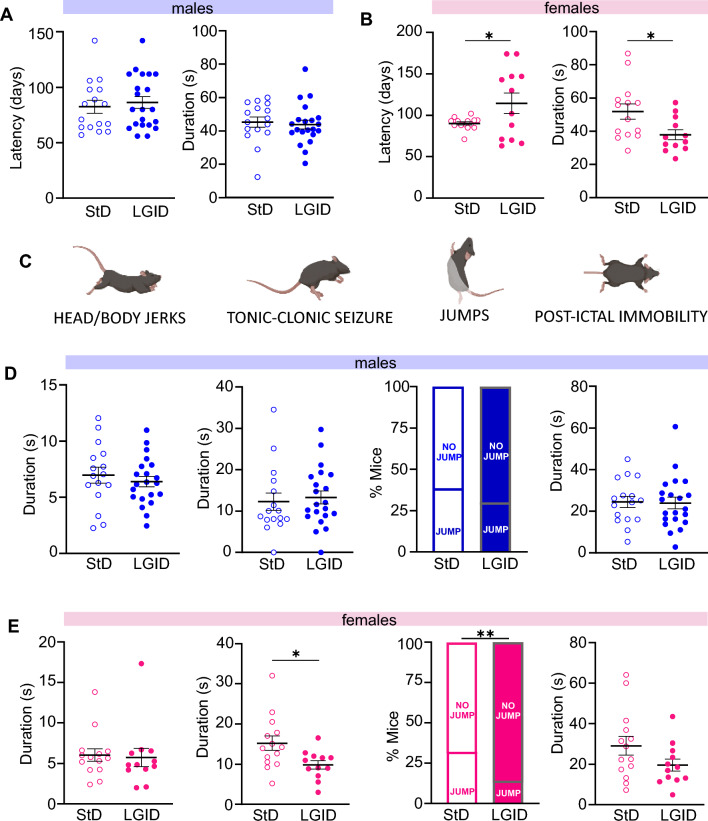
Analysis of the first observed seizure in male and female SynII KO mice. **A**, **B** Latency to the first seizure and its duration in males (**A**) and females (**B**). No differences in latency and duration of the first seizure were observed in males, while the seizure onset was delayed and of reduced duration in female mice treated with LGID. **C** Representative images of the main seizure behaviors analyzed: head and body jerks, tonic–clonic attacks, jumping and post-ictal immobility. **D**, **E** Behavioral analysis of the seizures, including (from left to right): duration of head and body jerks, duration of tonic–clonic attacks, percentage of animals jumping during seizures and duration of post-ictal immobility. These parameters revealed a significant decrease in the duration of the tonic–clonic attacks and in the percentage of animals jumping only in females treated with LGID. Data are expressed as means ± sem (*n* = 16 M StD; *n* = 21 M LGID; *n* = 14F StD; *n* = 12F LGID). *p < 0.05, **p < 0.01; unpaired Student’s *t*-test

Behavioral seizures were tested weekly, starting from the day of weaning, manipulating the mice in an isolated environment and moving them to a new cage, a procedure known to efficiently elicit reflex seizures in SynIIKO mice [30, 31]). The latency to the first seizure showed no differences between the two experimental groups (Supplementary Fig. 1A), while the total seizure duration was significantly shorter in SynIIKO mice treated with LGID (Supplementary Fig. 1B). All mice experienced generalized seizures initiated by a first short-lasting (5–15 s) phase of rapid muscle twitching affecting head, tail or legs (head and body myoclonus jerks, including retreating orofacial/forelimb twitch) (Supplementary Fig. 1C). The second phase consisted in typical tonic–clonic episodes with duration ranging between 5 and 35 s (Supplementary Fig. 1D) with jumps in about one third of the mice, generated by particularly intense clonic muscle contractions (Supplementary Fig. 1E). All seizures ended with a post-ictal immobility phase of variable duration, ranging from few seconds to one minute (Supplementary Fig. 1F). The various phases of the behavioral seizures were not affected by the diet when male and female data were pooled (Supplementary Fig. 1D–F). However, when male and female data were considered separately, a significant effect of LGID was observed only in females with longer latency to the first seizure and decrease of total seizure duration (Fig. 2A, B). Similarly, a significant reduction of the duration of the tonic–clonic phase and of the percentage of jumping mice was observed in female, but not in male mice (Fig. 2D, E). The gender-specific efficacy of LGID was not related to any sex-dependent difference in food or water consumption (Supplementary Fig. 2) or in the capability of LGID to decrease blood glucose or HbAc1 levels (Supplementary Fig. 3).

### Sex-dependent effects of LGID on neuronal excitability

I-IC discharges in cortico-hippocampal slices are considered a proxy of the epileptic phenotype [50] and have been already described in SynIIKO mice [37]. We investigated these bioelectrical markers of epilepsy in cortico-hippocampal slices obtained from symptomatic 5 months old SynIIKO mice of either sex treated with either LGID or StD. I-IC activity was analyzed by the HD-MEA system, that allows simultaneous extracellular recordings from 4096 electrodes at high spatial and temporal resolution (Supplementary Fig. 4A). The convulsant agent 4-AP (200 μM; [42]) was applied in the bath to favor the induction of epileptiform activity characterized by spontaneous spike-wave Inter-Ictal (I-IC) discharges. Cortical and hippocampal I-IC activity captured by the HD-MEA, can be visualized as a video, where each pixel of the video’s frames represents a recording-electrode of the array and the color of the pixel corresponds to the voltage amplitude detected by the recording pixel/electrode (Supplementary Fig. 4B). The spike-wave complex, recorded by each recording pixel/electrode (Supplementary Fig. 4C) is an “I-IC wave”, while a group of “I-IC waves” that are temporally aggregated and recorded by an ensemble of spatially clustered pixels, represents an “I-IC event” (Supplementary Fig. 4D).

**Fig. 3:**
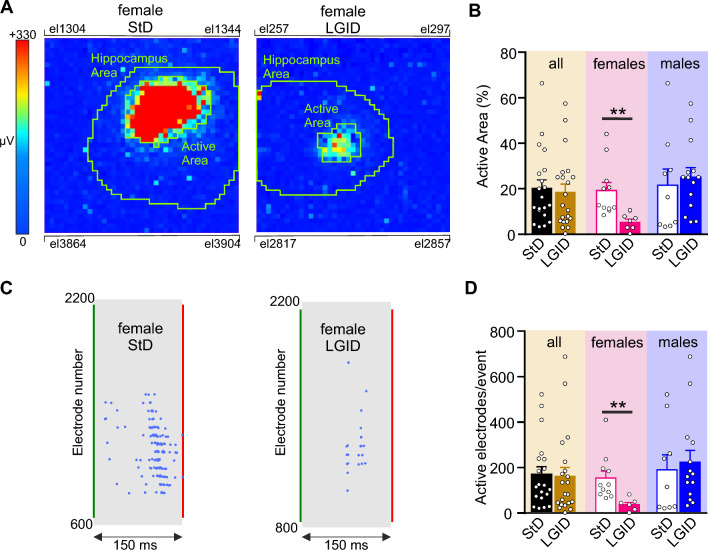
Spatial constraint of I-IC events in the hippocampus of SynIIKO female mice treated with LGID. **A** Two video frames showing color-coded maps of two I-IC events activated in the hippocampal slice of two 5-month-old female SynIIKO mice treated with either StD (right panel) or LGID (left panel). The green lines delimit the hippocampal area. Pixel size: 81 μm by side. **B** The bar plot shows means ± sem and individual values of the percentage areas of the hippocampus invaded by the I-IC events in all (left), female (center) and male (right) SynIIKO mice treated with either StD or LGID. **C** Raster plots of the two I-IC events shown in panel A. Single I-IC events recorded in the hippocampus of a female SynIIKO mouse treated with either StD (left) or LGID (right). Each blue dot represents I-IC waves recorded by the pixel-electrodes. The green and red lines represent the start and the end of the I-IC event, respectively. **D** The bar plot shows means ± sem and individual values of the number of electrodes activated during an I-IC event in hippocampal slice of all (left), female (center) and male (right) SynIIKO mice treated with either StD or LGID (*n* = 20 and 21 for StD- and LGID-treated Syn II KO mice; *n* = 11 and 7 for StD- and LGID-treated Syn II KO female mice; *n* = 9 and 14 for StD- and LGID-treated Syn II KO male mice, respectively). **p < 0.01; unpaired Student’s *t*-test

When all SynIIKO mice were compared, irrespective of sex, the percentage of hippocampal area invaded by an I-IC event, was not affected by the dietary condition. On the contrary, this parameter was dramatically reduced in LGDI-treated SynIIKO females and not affected in males treated with the same diet (Fig. 3A, B). Similar results were obtained when we quantified the number of electrodes activated during an I-IC event in the hippocampal field (Fig. 3C, D).

**Fig. 4:**
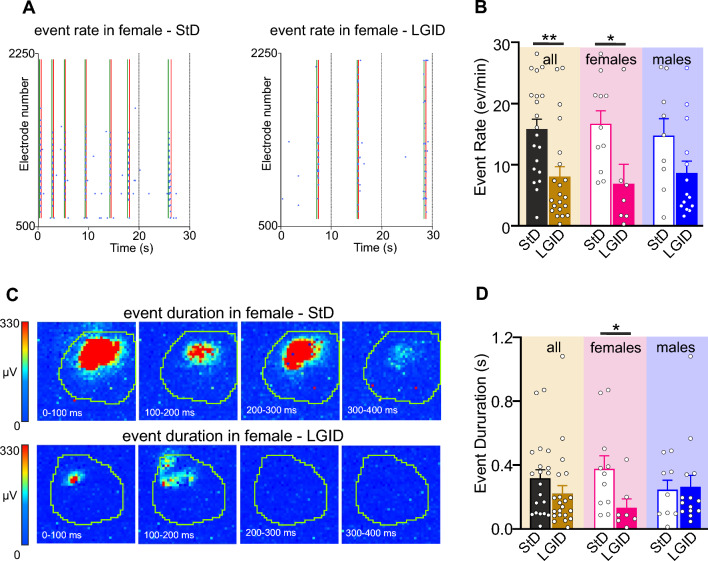
LGID reduces frequency and duration of hippocampal I-IC events in SynIIKO female mice. **A** Representative raster plots showing various I-IC events detected in a time window of 60 s in the hippocampus of two 5-month-old SynIIKO female mice treated with either StD (left panel) or LGID (right panel). The green and red lines represent the start and the end of the detected I-IC event, respectively. Each blue dot represents I-IC waves recorded by the pixel-electrodes. **B** The bar plot shows means ± sem and individual values of the I-IC event frequency in hippocampal slices from all (left), female (center) and male (right) SynIIKO mice treated with either StD or LGID. **C** The two series of video frames show color-code maps of two hippocampal I-IC events recorded in two 5-month-old SynIIKO female mice treated with either StD (upper panels) or LGID (lower panels). Each color-code map is computed as the variation of amplitude in 100 ms with the whole series that covers a time window of 500 ms. Pixel size: 81 μm by side. **D** The bar plot shows means ± sem and individual values of I-IC event duration in hippocampal slices of all (left), female (center) and male (right) SynIIKO mice treated with either StD or LGID (*n* = 20 and 21 for StD- and LGID-treated SynIIKO mice; *n* = 11 and 7 for StD- and LGID-treated female SynIIKO mice; *n* = 9 and 14 for StD- and LGID-treated male SynIIKO mice; respectively). *p < 0.05, **p < 0.01; unpaired Student’s *t*-test

The frequency of hippocampal I-IC events was significantly reduced in the whole population of LGID-treated SynIIKO mice, regardless of sex. However, when this parameter was compared in sex matched mice, the reduction of I-IC rate induced by LGID remained significant only in females (Fig. 4A, B). Similarly, when the mean duration of hippocampal I-IC events was assessed in sex-matched or unmatched SynIIKO mice, LGID induced a significant and marked decrease of I-IC duration only in females (Fig. 4C, D).

**Fig. 5:**
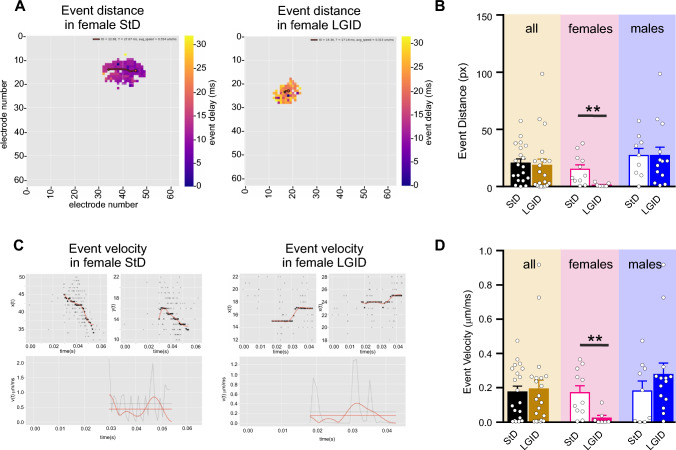
LGID reduces covered distance and propagation speed of hippocampal I-IC events in SynIIKO female mice. **A** The two video frames represent color-coded delay maps of two 5-month-old SynIIKO female mice treated with either StD (left panel) or LGID (right panel). The trajectory paths (in red) plotted over the delay map are used to calculate the distance covered by each I-IC event. The filled circle corresponds to the starting point of the trajectory. **B** The bar plot shows means ± sem and individual values of the distance covered by the I-IC event in hippocampal slices of all (left), female (center) and male (right) SynIIKO mice treated with either StD or LGID. **C** Upper panels show track trajectories of each I-IC event on the bidimensional plane of the chip, decomposed in its horizontal and vertical components, for two representative 5-month-old SynIIKO female mice treated with either StD (left panels) or LGID (right panels). Lower panels show the propagation speed (red horizontal line) of the I-IC event, calculated by averaging the smoothing (smooth red line) of instantaneous speed (gray jagged line) of each trajectory for two female mice treated with either StD (left panel) or LGID (right panel). **D** The bar plot shows means ± sem and individual values of the propagation speed of the I-IC events in hippocampal slices of all (left), female (center) and male (right) SynIIKO mice treated with either StD or LGID (*n* = 20 and 22 for StD- and LGID-treated SynIIKO mice; *n* = 11 and 7 for StD- and LGID-treated female SynIIKO mice; *n* = 9 and 14 for StD- and LGID-treated male SynIIKO mice; respectively). **p < 0.01; unpaired Student’s *t*-test

**Fig. 6:**
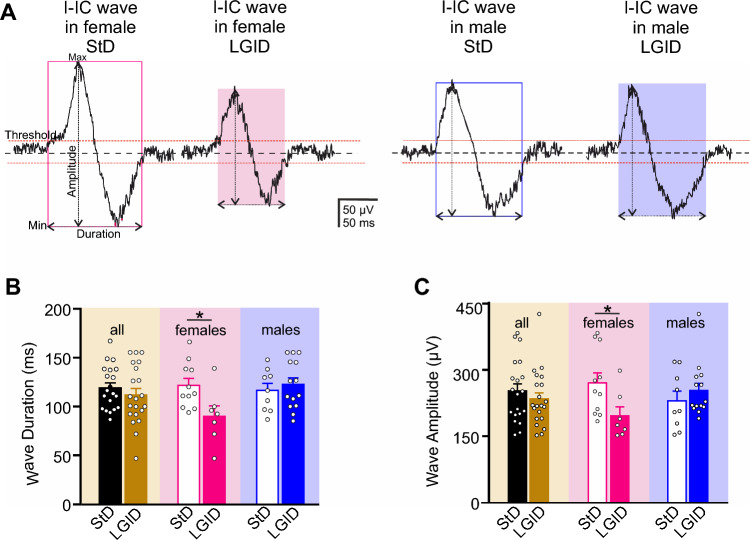
LGID reduces duration and amplitude of hippocampal I-IC waves in SynIIKO female mice. **A** Representative electrophysiological traces of I-IC waves recorded in the hippocampus of 5-month-old SynIIKO female (left) and male (right) mice treated with either StD or LGID as indicated. **B** The bar plot shows means ± sem and individual values of the I-IC wave duration in hippocampal slices of all (left), female (center) and male (right) SynIIKO mice treated with either StD or LGID. **C** The bar plot shows means ± sem and individual values of the I-IC wave amplitude in hippocampal slices of all (left), female (center) and male (right) SynIIKO mice treated with either StD or LGID (*n* = 20 and 21 for StD- and LGID-treated SynIIKO mice; *n* = 11 and 7 for StD- and LGID-treated female SynIIKO mice; *n* = 9 and 14 for StD- and LGID-treated male SynIIKO mice; respectively). *p < 0.05; unpaired Student’s *t*-test

We also estimated the covered distance and propagation speed of each I-IC event idealized to a single “center of mass”. Also in this case, the distance and the velocity of the I-IC events were dramatically reduced by LGID treatment, but only in the female SynIIKO group (Fig. 5). Similar results were obtained when we measured the duration and amplitude of the spike-wave complexes (“I-IC waves”; Fig. 6A) that were again significantly reduced in duration and amplitude only in female SynIIKO mice treated with LGID (Fig. 6B, C).

HD-MEA recordings of cortical epileptiform activity highlighted a reduced efficacy of LGID with respect to the effects observed in the hippocampus. LGID was only effective in reducing the event rate, the event duration and the duration of the I-IC waves regardless of sex (Supplementary Fig. 5). However, when these parameters were compared in sex matched mice, the reduction of event and wave duration remained significant only in females, while it was lost in the male group.

### Sex-dependent effects of LGID on cortico-hippocampal allopregnanolone levels

**Fig. 7:**
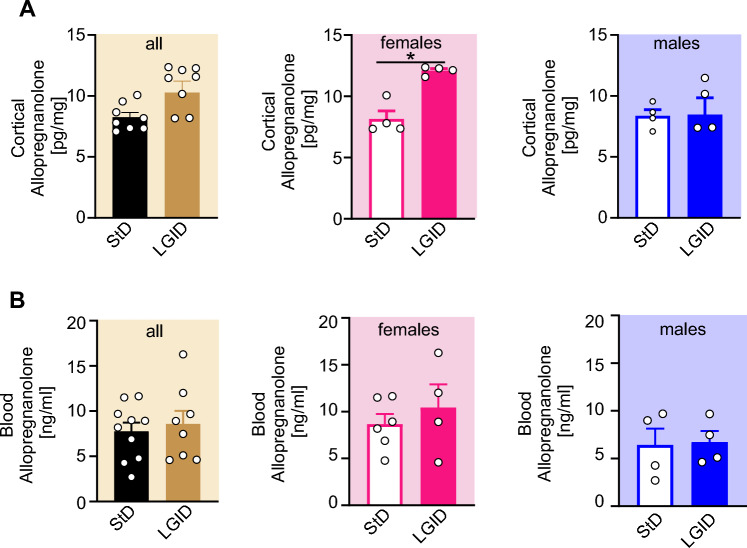
LGID increases cortical ALLO concentration in female Syn II KO mice but not in male. **A**, **B** The bar plots show means ± sem and individual values of ALLO concentrations in the cortico-hippocampal tissue (**A**) and plasma (**B**) of all (left), female (center) and male (right) SynIIKO mice fed either StD or LGID (*n* = 8 and 8 for both StD- and LGID-treated SynIIKO mice; *n* = 4 for both StD- and LGID-treated female SynIIKO mice; *n* = 4 for both StD- and LGID-treated male SynIIKO mice). *p < 0.05; unpaired Mann–Whitney *U*-test

Both behavioral and electrophysiological data revealed a gender-specific protective action of LGID limited to SynIIKO females, suggesting a possible modulation of dietary treatment on the sex-related neurosteroid pathway. We focused our investigation on ALLO, well known for its anti-seizure action ascribed to a potent allosteric modulation on GABA_A_ receptors [51, 52]. ELISA-based analysis on extracts of cortico-hippocampal tissue revealed an increase of ALLO concentration only in LGID-fed females, while the same effect was not present in LGID-treated males (Fig. 7A). This effect was only present within the brain, as LGDI did not affect plasma ALLO levels regardless of gender (Fig. 7B).

## Discussion

Despite decades of research activity, currently there is no FDA-approved treatment that truly prevents the development of epilepsy in people at risk. The negative results obtained using AEDs strongly impose a switch towards new strategies of intervention [4, 6, 53–55]. Most of the AEG*-*trials with AEDs aimed at preventing epilepsy following traumatic brain injury or stroke were unsuccessful also due to the heterogeneity of the patient populations [56]. The investigation of new strategies for the prevention of epilepsy could probably take advantage from the phenotypic homogeneity that characterizes genetic models of epilepsy, in which seizures occur either spontaneously or in response to sensory stimuli. The numerous genetic animal models of epilepsy characterized over the recent decades [57] have the advantage to simulate the vast majority of “idiophatic” epilepsy syndromes more closely than any other experimental model of epilepsy [58]. For these reason, to investigate the AEG action of LGID, we chose the SynIIKO mouse, a human monogenic epileptic synaptopathy, whose epileptogenic process was extensively characterized by us and others [25]. In this mouse, the deletion of SynII induces upregulation of synchronous release of GABA and a concomitant loss of delayed asynchronous release that are already present in pre-symptomatic mice. The lack of asynchronous GABA release impairs tonic inhibition due to the activation of GABAergic extra-synaptic receptors, in turn leading to an augmented firing activity at both single neuron and network levels [37, 59].

The history of dietary therapy for epilepsy is quite long, already Hippocrates documented the use of caloric restriction to treat epilepsy [60]. The ketogenic diet (KD) has been employed as a treatment for drug-resistant epilepsy for over 90 years [13]. Despite the substantial efficacy [61, 62], the use of KD remains limited because of difficulties in implementation and tolerability. Recently, neuroscientists have proposed several antiepileptic treatment methods that involve metabolic regulation [15, 63, 64]. An effective alternative dietary approach is the LGID, which in many cases showed an efficacy comparable to the classic KD, but it is much better tolerated [9–12, 65, 66]. For this reason, LGID may better respond to the need of long-term preventive therapy to contrast epileptogenesis in healthy patients, often children, with high probability to develop epilepsy.

Here, we tested in vivo and ex vivo effects of the early application of LGID in SynIIKO mice starting from the prenatal phase. Behavioral characterization revealed that LGID produces a significant delay in the appearance of the first seizure and a decrease of its duration. Surprisingly, this effect was observed only in females. The ex vivo electrophysiological investigation confirmed the gender-dependent sensitivity by showing a decrease of evoked epileptic-like activity only in female SynIIKO mice fed with LGID. This specific effect was not attributable to any gender-related differences in food/water intake, body weight, blood glycemic index or glycated hemoglobin concentration.

What could be the mechanism of action for antiepileptogenic effect of LGID? Although dietary restrictions are widely used for the treatment of drug-resistant epilepsies, their mechanisms of action are still under investigation [5, 16]. Various studies have highlighted that the decrease of neuronal glucose utilization, obtained by different low-glucose diets such as KD, LGID or modified Atkins diet or, alternatively, by glycolytic pathway inhibitors, such as 2-deoxy-glucose (2DG) represents the common mechanism at the basis of the antiepileptic action [14]. This hypothesis is further strengthened by the evidence that hyperglycemia lowers seizure threshold [5, 67] and infusion of glucose in patients under KD treatment results in restoration of the seizures [68]. Moreover, the anti-diabetic drug metformin is now recognized as a new potential AED or even AEG drug [69].

During the last 2 decades, at least three distinct mechanisms for the anti-seizure effects of low-glucose diets have been identified [70]. It was initially reported that reduced glucose availability interferes with the membrane conductance of neurons, primarily via ATP-sensitive potassium (K_ATP_) channels that act as metabolic sensors coupling neuronal excitability to ATP levels [63, 71, 72]. Indeed, a reduction of cytoplasmatic ATP concentration has a hyperpolarizing effect mediated by K_ATP_ channel opening [73]. More recently, it was shown that the transcriptional repressor REST/NRSF is activated by the reduced intracellular concentrations of NADH induced by the inhibition of glycolysis [15]. Such metabolic recruitment of REST/NRSF can induce the transcriptional repression of BDNF and its receptor TrkB [15] and activate other homeostatic pathways [74]. Indeed, REST/NRSF expression and translocation to the nucleus reduces neuronal firing [75], scales down excitatory inputs and potentiates inhibitory transmission onto excitatory neurons [76, 77]. More recently, it was also shown that REST/NRSF boosts K^+^ buffering and glutamate reuptake in astrocytes that are critical to maintain synaptic homeostasis [78].

Finally, several groups reported that 2DG-induced inhibition of glycolysis favors the synthesis of NADPH by enhancing the pentose phosphate pathway (PPP) in neurons [79–82]. PPP is the major source of NADPH in neurons [83] and is enhanced not only by 2DG, but also in case of reduced brain glucose availability [79], fasting [84, 85] or KD [86]. In these cases, glucose is redirected to the PPP, as shown by the increase of PPP metabolites, probably due to the increased demand for NADPH, aimed at strengthening antioxidant defense. NADPH acts as the crucial co-factor of 5α-reductase (5α-R), the rate-limiting enzyme for ALLO biosynthesis [52, 87]. The N-terminal part of 5α-R binds to steroid substrates, whereas the C-terminal portion containing a glycine-rich region binds NADPH [88]; consequently, a higher concentration of NADPH will increase neuronal ALLO production [89]. Also known as endogenous benzodiazepine, ALLO acts as a potent allosteric modulator of synaptic and extrasynaptic GABA_A_ receptors and, therefore, enhances both phasic and tonic inhibition [90–92]. In particular, tonic inhibition, mediated by extrasynaptic GABA_A_ receptors bearing α/δ subunits is specifically sensitive to neurosteroids, and the resulting potentiation of tonic conductance favors a form of shunting inhibition that strictly controls neuronal network excitability and seizure susceptibility [93].

Given the pro-epileptogenic effect of the lack GABAergic tonic inhibition identified in SynIIKO mice [37, 59], we focused on the increase in brain ALLO levels as the potential mechanism for the anti-epileptogenic action of LGID and for its gender-specificity. We observed similar ALLO concentrations in the brain of male and female SynIIKO mice fed with StD, as previously reported in the brain of naïve rodents [94–96]. Notably, LGID treatment increased ALLO levels only in female cortico-hippocampal area, whilst the plasmatic concentration of ALLO was not affected by LGID in both sexes. These results suggested that the gender-specificity of LGID is probably not related to changes of peripheral levels of ALLO but could derive from a sex-specific increase of local synthesis of ALLO in the brain of SynIIKO females fed with LGID.

Our data on cortical ALLO, although still preliminary, indicate an avenue for future research. To find a mechanistic explanation for the LGID-induced increase of cortical ALLO production in females, it will be crucial to consider that: (i) female rodents have plasma and cortical progesterone (PROG) concentrations higher than males, irrespective of the phase of their estrous cycle [95, 97] and (ii) ALLO is synthesized in the brain from PROG by the sequential action of 5α-R, which reduces PROG to 5α-dihydroprogesterone (5α-DHP) and 3α-hydroxysteroid oxidoreductase (3α-HSOR), which converts 5α-DHP into ALLO [98]. Thus, PROG is the precursor of the enzymatic cascade, initiated and rate-limited by 5α-R, that leads to ALLO biosynthesis in the brain [52].

Thus, future research activity will have the task of investigating whether, in females fed with LGID, the increase of NADPH concentrations potentiates the 5α-R activity and ALLO biosynthesis thanks to the higher PROG availability, an effect that in males can be greatly reduced or absent because of the limited availability of PROG.

Other causes of the gender specificity, that deserve to be taken in consideration, are the possible sex differences in 5α-R activity and/or in the sensitivity of GABA_A_ receptors to ALLO. Indeed, it was previously shown that the brain of female green anole lizards expresses higher levels of 5α-R than males [99] and that female mice are more sensitive to the anti-epileptic effects of ALLO because of a greater abundance of δ-subunit containing extra-synaptic GABA_A_ receptors [100].

## Conclusions

The prevention of epilepsy is a relevant scientific challenge and an urgent unmet need. In human genetic epilepsy, no treatment is available thus far able to prevent the development of epilepsy in patients at risk [3]. The investigation of the functional mechanisms underlying the homeostatic processes activated by the treatment with LGID represents a step forward for the identification of novel therapeutic solutions. The efficacy of LGID in delaying seizure onset in female mice suggest that this highly sustainable diet-based therapy is a promising strategy not only as an antiepileptic treatment, but to prevent or delay the appearance of the epileptic phenotype in syndromes with distinct etiology sharing similar evolution of the epileptogenic process. Finally, the greater protection of LGID observed in females, underlines the importance of developing personalized gender-specific treatments.

### Supplementary Information

Below is the link to the electronic supplementary material.

Supplementary file1 (DOCX 922 KB)

## Data Availability

The datasets generated and analyzed during the current study are available from the corresponding authors on reasonable request.
