# Supplementary material for: Low glycemic index diet restrains epileptogenesis in a gender-specific fashion
Source: Cell Mol Life Sci. 2023 Nov 10;80(12):356. doi: 10.1007/s00018-023-04988-1 (PMC10638170; doi:10.1007/s00018-023-04988-1)
Supplement: Supplementary file 1 — Supplementary file1 (DOCX 922 KB) [file 18_2023_4988_MOESM1_ESM.docx]

**
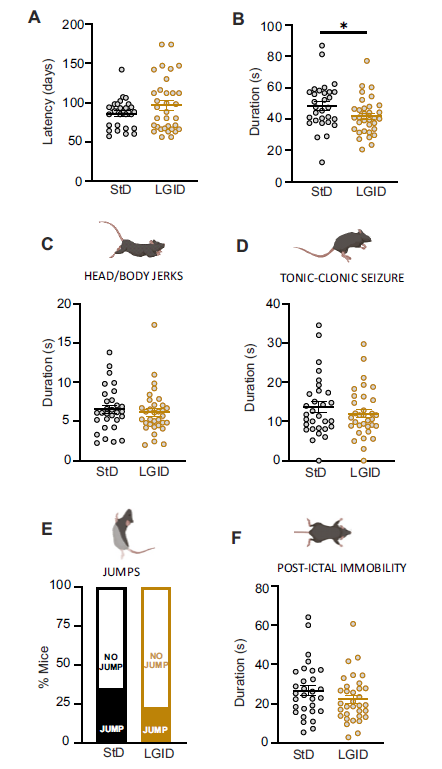
**

**Supplementary Figure 1. *Behavioral analyses of the first observed seizure.*** (**A**) Latency to the first seizure. The monitoring of the appearance of the first behavioral seizure showed no differences between the two experimental groups in the latency to seizure onset. (**B**) Duration of the first behavioral seizure. Mice treated with LGID displayed a shorter duration of the behavioral seizure when compared to StD-fed mice. (**C-F**) Representative images of the main seizure behaviors (*top*) and behavioral analysis of the main seizure elements (bottom) including: duration of head and body jerks (**C**), duration of the tonic-clonic attacks (**D**), percentage of animals jumping during seizure (**E**) and duration of post-ictal immobility (**F**). The analyzed parameters revealed no differences between the two experimental groups. Data are expressed as means ± sem (n=30 StD; n=33 LGID). *p < 0.05; unpaired Student's *t*-test.


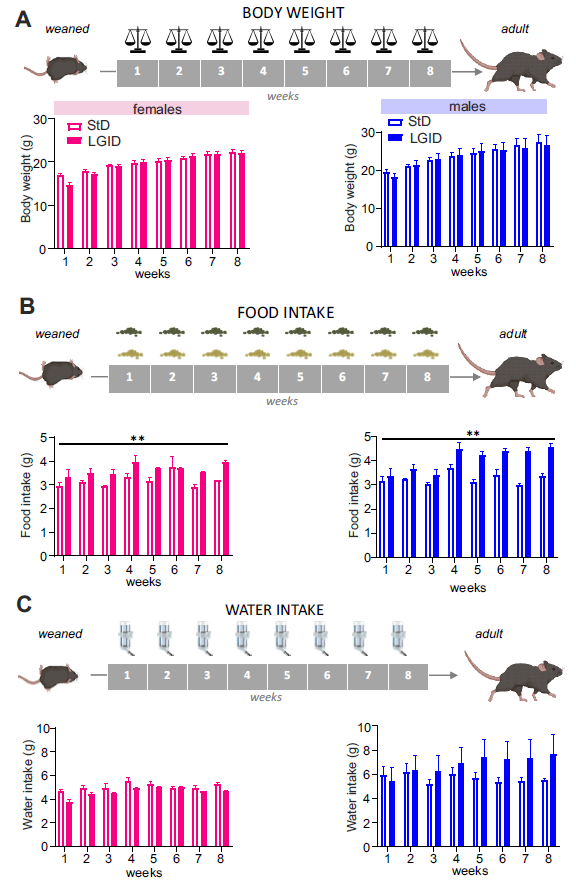


**Supplementary Figure 2 - Parameters analyzed during the administration of the two diets to male and female SynIIKO mice.** (**A**) Either StD or LGID were delivered *ad libitum* to SynIIKO pregnant females starting from mating and continuing during pregnancy and lactation. Mice were maintained with the same diet also after weaning and for the entire lifespan. Starting from the day of weaning, all mice were weekly checked for two months for body weight (**A**), food intake (**B**) and water consumption (**C**). Both male and female treated with LGID increased the food consumption in the presence of comparable body weight and water consumption (n=16M StD; n=21M LGID; n=14F StD; n=12F LGID). **p < 0.01; two-way repeated-measures ANOVA.


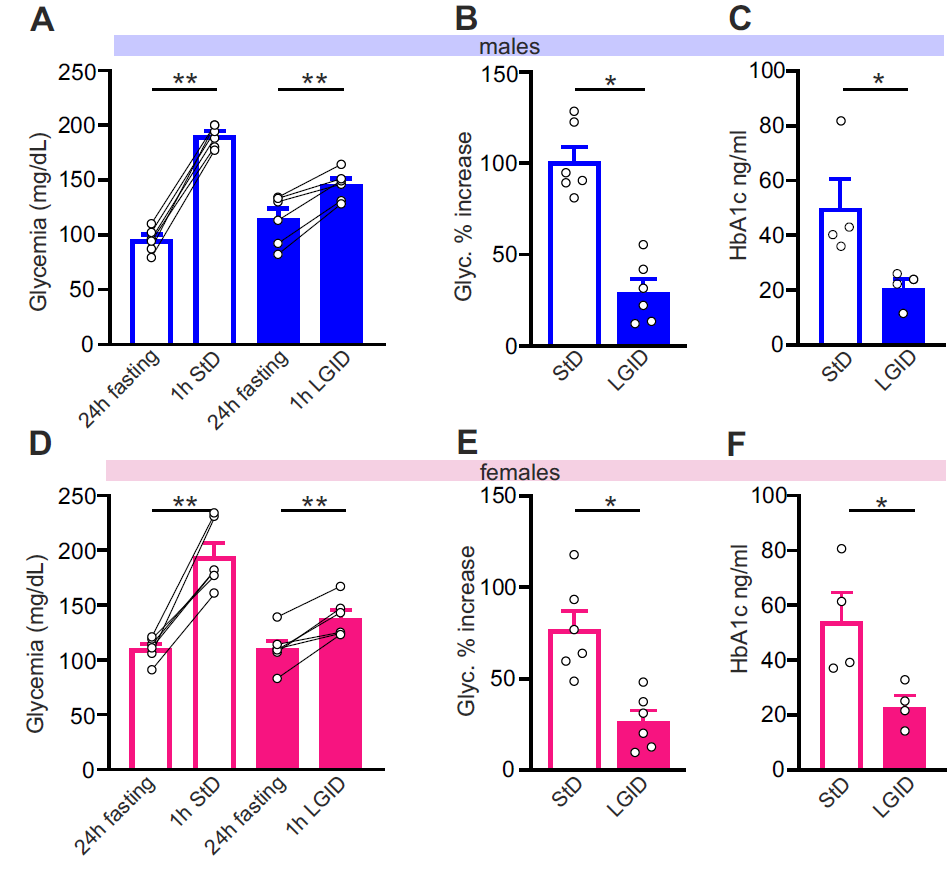


**Supplementary Figure 3.** ***LGID reduces glycemia and glycated hemoglobin in both male and female SynIIKO mice.*** At adult stage (5 months of age), male (**A-C**) and female (**D-F**) mice were subjected blood analysis. (**A,D**) Glycemia after a fasting period of 24 h and 1 h after food administration analysis. (**B,E**) Percent glycemic increase 1h after food intake. (**C,F**) Glycated hemoglobin levels. Bar plots show means ± sem and individual values (n=12 for both StD- and LGID-treated SynIIKO mice; n=6 for both StD- and LGID-treated female SynIIKO mice; n=6 for both StD- and LGID-treated male SynIIKO mice). Blood glucose levels and glycated hemoglobin levels showed significant increases after food consumption only in StD-fed mice with no sex-related differences. *p < 0.05, **p < 0.01; paired Wilcoxon’s test (**A,D**); unpaired Mann-Whitney *U*-test (**B,C,E, F**).


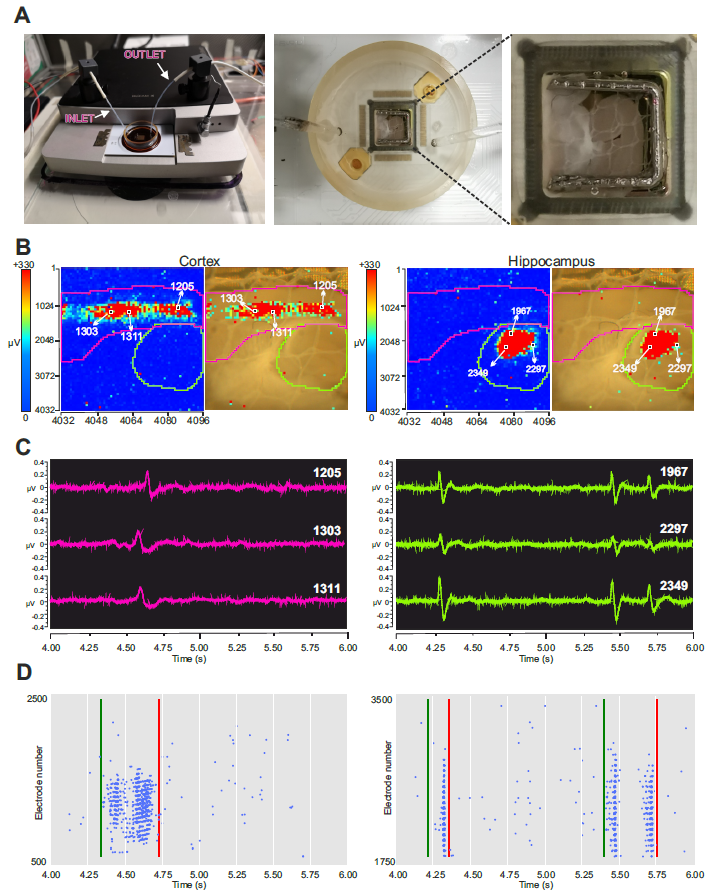


**Supplementary Figure 4**. ***Functional imaging of cortico-hippocampal slices****.* (**A**) *Left:* Image of the Biocam X, the HD-MEA system (3Brain AG), mounting a CMOS-based microelectrode array with 4096 platinum-coated recording electrodes. Note on the top of the Biocam X, the magnetic perfusion holder that keeps in place the perfusion tubing system. *Middle:* Close-up of the microelectrode array (model: Stimulo) recording chamber showing two glass capillaries, the inlet and the outlet of the perfusion system. *Right:* Higher magnification close-up of the sensing area (5.12 mm x 5.12 mm) large enough to record electrophysiological activity from the whole cortico-hippocampal slice. (**B**) A cortico-hippocampal slice, stabilized by a square-shape platinum anchor, over the active area of the chip. Video frame in pseudo-colors computed as the variation of amplitude in the 100 ms time window. The superimposed color-coded LFP activity shows spontaneous *I-IC events* invading large portions of cortical (*left*) and hippocampal regions (*right*). Each pixel of the frame represents an electrode of the array and the color of the pixel corresponds to the voltage amplitude recorded by the electrode, according to the color-code map on the left of the frame. Pixel size: 81 μm by side. (**C**) Representative electrophysiological traces of *I-IC waves* recorded by 3 electrodes (1205, 1303, 1311) located in the cortical area (*left*) and by 3 electrodes (1967, 2297, 2349) in the hippocampal area (*right*). (**D**) Representative raster plots of the two *I-IC events* shown in panel B. The green and red lines represent the start and the end of the *I-IC event,* respectively. In the left panel, a single *I-IC event* invading a large fraction of the cortical area is shown during a 2-s time window. In the right panel, two *I-IC events* recorded in the CA1 area of the hippocampus are shown, during the same time window. Each blue dot in the raster plot represents *I-IC waves* recorded by many pixel-electrodes.


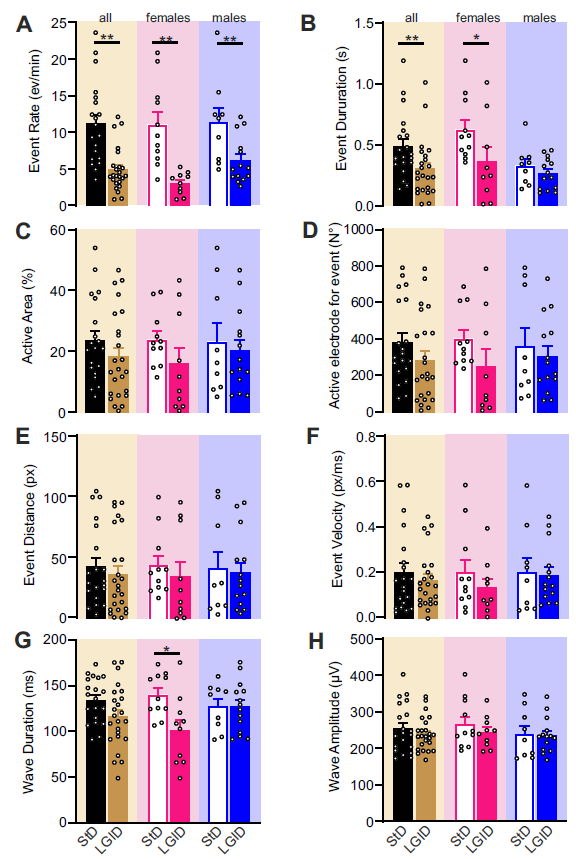


**Supplementary Figure 5. LGID treatment induces a moderate reduction of I-IC activity in the cortex of SynIIKO mice.** (**A-H**) Bar plots show means ± sem and individual values of rate (**A**), duration (**B**), percent area (**C**), active electrode (**D**), distance (**E**) and velocity (**F**) of cortical *I-IC events,* as well as duration (**G**) and amplitude (**H**) of cortical *I-IC waves* recorded in cortico-hippocampal slices of all (*left*), female (*center*) and male (*right*) SynIIKO mice treated with either StD or LGID. n=20 and 24 for StD- and LGID-treated SynIIKO mice; n=11 and 10 for StD- and LGID-treated female mice; n=9 and 14 for StD- and LGID-treated male mice; respectively. *p<0.05, **p<0.01; unpaired Mann-Whitney *U*-test (**A-F**) or unpaired Student’s *t*-test (**G,H**).

|  | **LGID** | **StD** |
| --- | --- | --- |
| **Ingredient** | *g/kg* | *g/kg* |
| Corn starch Amioca | 0 | 542 |
| Corn starch Hylon VII | 542 | 0 |
| Gelatine | 20 | 20 |
| Casein | 200 | 200 |
| DL Methionine | 2 | 2 |
| Vitamin mix | 10 | 10 |
| Mineral mix | 35 | 35 |
| Sucrose | 85 | 85 |
| Wheat bran | 50 | 50 |
| Soybean oil | 56 | 56 |
| Total | 1000 | 1000 |

**Supplementary Table 1:** Composition of LGID and StD. The amounts of each ingredient are given in g/kg of food.
